# Sphenoid Inverted Nasal Papilloma Presenting as Chronic Headaches in a 21-Year-Old Patient: A Case Report and Literature Review

**DOI:** 10.7759/cureus.65262

**Published:** 2024-07-24

**Authors:** Claudia Gonzalez, Isamar Fernandez, Jordy Batista, Diana Durán, Massiel Gonzalez

**Affiliations:** 1 School of Medicine, Pontificia Universidad Católica Madre y Maestra, Santiago, DOM; 2 School of Medicine, Universidad Iberoamericana (UNIBE), Santo Domingo, DOM; 3 Department of Radiology, Diagnostica Social -Patronato Benéfico Oriental (PBO), La Romana, DOM

**Keywords:** recurrence, case report, inverted papilloma, schneiderian papilloma, sphenoid sinus

## Abstract

An inverted papilloma is a rare, benign tumor that affects the nasal cavity and paranasal sinuses. The maxillary and ethmoid sinuses are the most commonly affected, while the involvement of the sphenoid sinus is rare and may be associated with malignancy. We describe the case of a 21-year-old female who presented with recurring headaches along with dizziness, difficulty concentrating, mild hypoacusis, and occasional nasal congestion. A CT of the sinuses showed a soft tissue lesion in the sphenoidal sinus with extension into the posterior ethmoidal cells. After the biopsy, the patient was ultimately diagnosed with sphenoid inverted nasal papilloma.

## Introduction

Sinonasal papillomas, also called Schneiderian papillomas, are a group of benign neoplasms derived from the respiratory mucosa on the skull cavities. These tumors represent 0.5%-4% of all sinonasal tumors [1]. The World Health Organization (WHO) divides sinonasal papillomas into three types: inverted, exophytic, and oncocytic [2]. Inverted papilloma mainly affects the nasal cavity and paranasal sinuses. The most commonly affected are the maxillary and ethmoid sinuses. The sphenoid sinus involvement is infrequent and sometimes associated with recurrence and malignancy [3]. It presents with nonspecific signs and symptoms such as unilateral nasal obstruction, rhinorrhea, epistaxis, etc. However, inverted papillomas of the sphenoid sinus have headaches as the most common sign [4]. It represents a high recurrence rate, from 20% to 47% of cases, and is most frequently observed in the fifth and sixth decades of life [2,4]. 

## Case presentation

**Figure 1:**
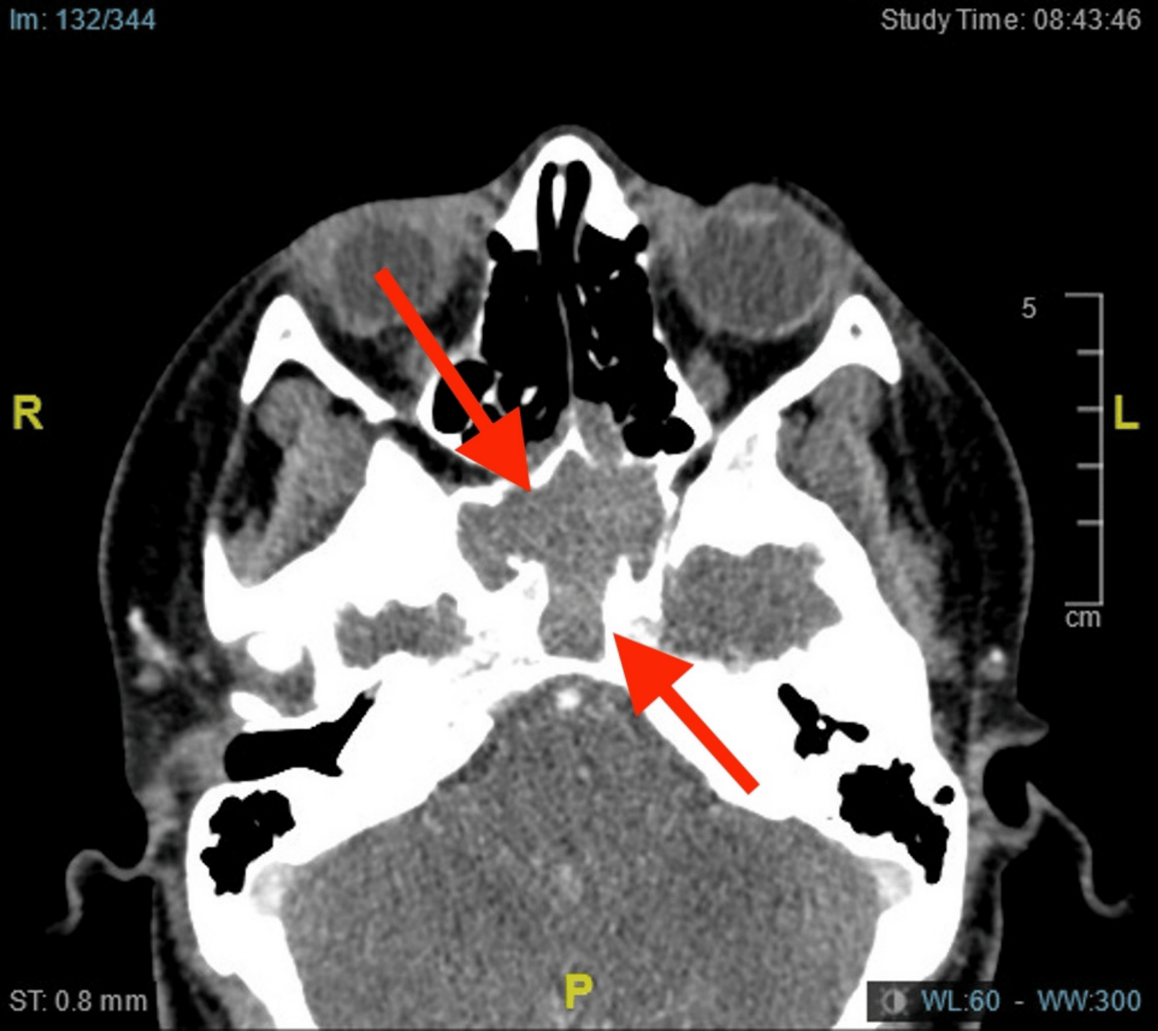
In the CT scan of the paranasal sinuses, hypodensity of soft tissue was identified, with occupation of the sphenoid sinus extending to the ostium of the sphenoid sinus, associated with bone erosion and partial destruction of the clivus (red arrow).

A 21-year-old female patient was referred to the otorhinolaryngology consult for a two-year history of recurring headaches along with dizziness, difficulty concentrating, mild hypoacusis, and occasional nasal congestion that did not resolve with pharmacological treatment. During the consult, the patient described constant headaches with an intensity rating of 9/10, radiating to the neck and periorbital area. Moreover, persistent thick and clear nasal discharge was observed. A CT scan of the sinuses showed an opacifying lesion in the sphenoidal sinus with focal extension into the posterior ethmoidal cells, resulting in bone thinning between the sphenoidal and posterior ethmoidal portions. Additionally, adenoid tissue growth, hypertrophic turbinates, and right septal dysmorphia were reported (Figure 1).

**Figure 2:**
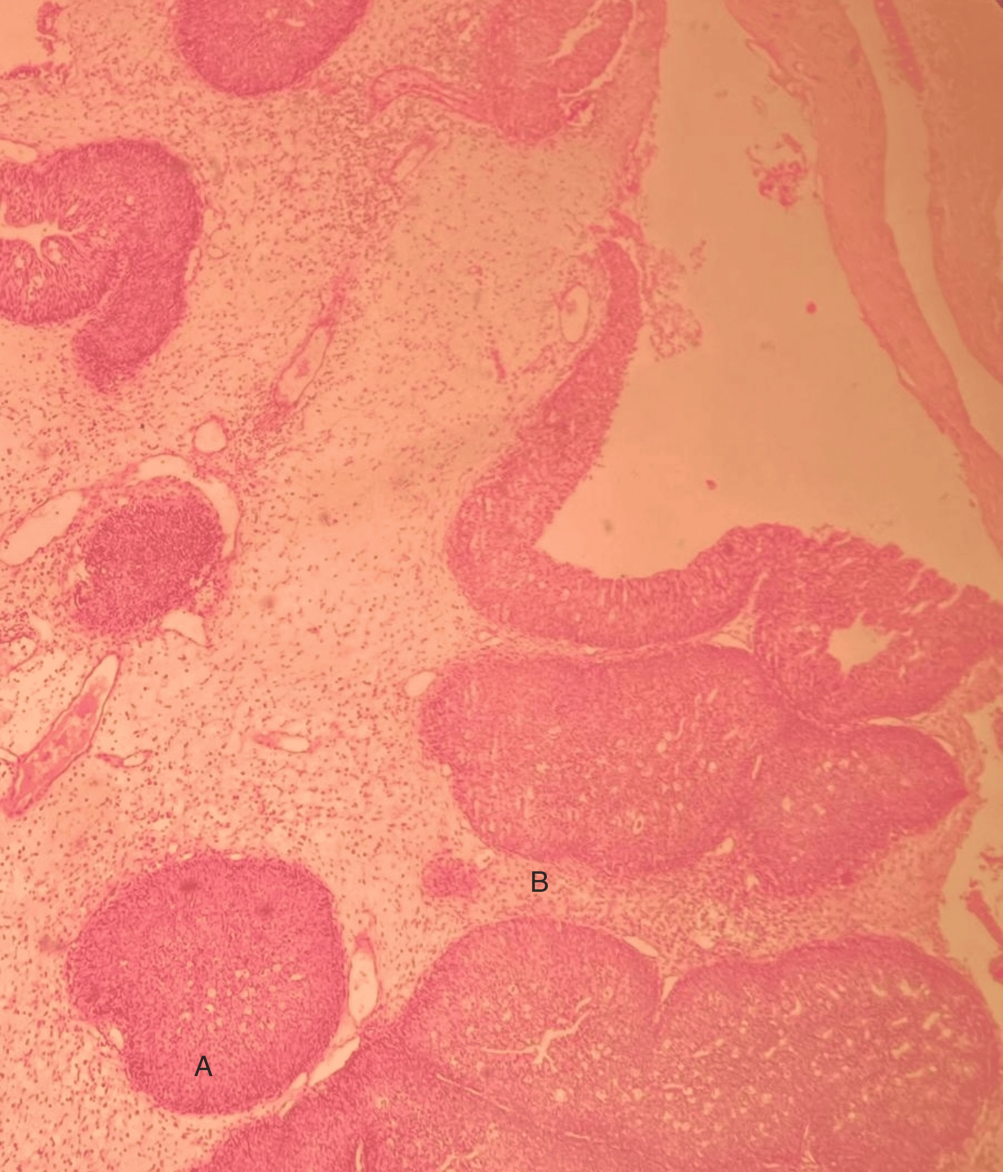
Microscopic description of the excised tissue revealed growth with a central fibrovascular stalk covered by pseudostratified epithelium. In the lamina propria, edema, congestion, and abundant mononuclear cells were seen. As a result, nasal mucosa and nasal turbinate resection products indicated portions of inverted nasal papilloma associated with edematous respiratory mucosa and chronic inflammation.

Based on these findings, the patient underwent bilateral maxillary antrostomy (Caldwell-Luc procedure), endoscopic anterior and posterior ethmoidectomy, and endoscopic sphenoidectomy. Resected tissue was sent for histological analysis. Pathology received fragments of the sphenoid and ethmoid bone and several irregular fragments of soft, whitish tissue, measuring 3.5 x 3 x 1.2 cm. Microscopic evaluation confirmed the diagnosis of sphenoid inverted nasal papilloma (Figure 2).

Follow-up and outcomes

Ten days after surgery, the patient affirmed the disappearance of headaches, nasal congestion, dizziness, and hypoacusis. A follow-up CT scan two months after the surgery revealed no abnormalities. 

**Figure 3:**
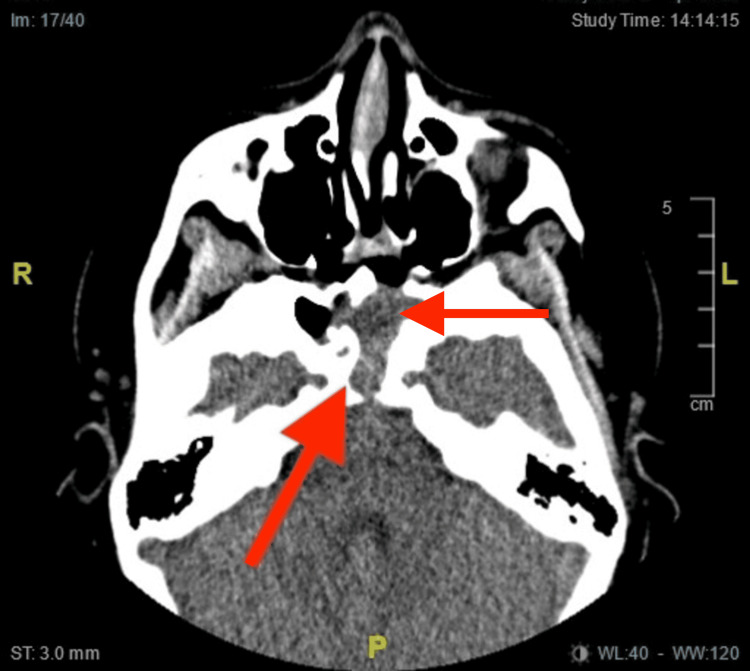
In the CT scan of the paranasal sinuses, postsurgical changes due to sphenoidotomy were identified with enlargement of the sphenoid ostium. Hypodensity of soft tissue was noted, with partial occupation of the sphenoid sinus extending to the sphenoid ostium. This was associated with bone erosion and destruction of the clivus, extending to the posterior wall of the clivus (red arrow).

Despite appropriate treatment, eight months later the patient returned to consult because of the recurrence of nasal congestion and headaches. A new CT revealed mucosal occupation at the level of the sphenoidal sinus associated with periosteal reaction and evident bone erosion in the posterior wall of the clivus and the inferior wall of the sphenoid bone, supporting the diagnosis of recurrent nasal papilloma (Figure 3).

A second surgical intervention successfully removed the lesion. Microscopic evaluation of the specimen confirmed the recurrence of inverted papilloma.

## Discussion

Inverted papilloma is characterized by histopathology of endophytic growth into the basal membrane, forming interconnected epithelial nests [5]. They are the most common subtype of Schneiderian papillomas (47%-78%) [1,6], with a male-to-female ratio of 2-5:1 [1, 7], and are more commonly found through the fourth and seventh decade of life [1]. Inverted papilloma most commonly presents with progressive unilateral-though it can be bilateral in some cases-nasal obstruction followed by rhinorrhea, epistaxis, and facial pain due to sinus obstruction. [5, 6, 8]. Due to inverted papilloma's unlimited growth potential leading to morbidity and considerable risk for malignant transformation (~10%) [9], surgical resection is the best approach for treatment. The technique of choice will depend on the size and location of the lesion, with options ranging from radical lateral rhinotomy to snare avulsion. However, regardless of the technique used, recurrence/persistence rates are high (recurrence of 9.7%-60%, average 30.9%) [10, 11].

Based on a literature review of nine cases regarding inverted papilloma, six (66.6%) of the patients were male and had a mean age of 49.5, ranging from 11 to 77 years. Of these patients, three of them were less than 40 (33.3%), and only one was 18 years or younger. Most cases of inverted papilloma present between the fifth and eighth decade of life, but there are some rare cases in which it can present before this range. There can also be exceptional cases where inverted papilloma can be identified in the third decade, as observed in this one.

Regarding the original location of the inverted papilloma [11-19], five of the cases originated from the nasal cavity (55.5%); two from the lacrimal sac or duct (22%); one from the middle ear cavity; and one from the maxillary sinus. Out of all of these, two of them extended to nearby cavities such as the ethmoidal and frontal sinus, mastoid antrum, and orbital cavity. The two most common symptoms on presentation were nasal obstruction and discharge, accounting for four (44%) of the cases. Other symptoms related to the location of the mass included epiphora, hipoacusis, otorrhea, and anosmia. Out of these, four of the cases (44%) were a recurrent lesion with prior surgical excision, as was in the one presented here.

## Conclusions

In conclusion, the presented case of inverted sinonasal papilloma in a young female deviates from the typical epidemiological standard, highlighting the variable presentation of this tumor. This case brings to attention the importance of recognizing the variable nature of sinonasal papillomas (location and clinical manifestations) for their prompt diagnosis and treatment. It should be noted that due to its high recurrence rate, long-term follow-up is paramount for the management of this condition. Future research should focus on identifying early diagnostic markers and optimizing treatment protocols to manage such atypical presentations.
